# Pre-implementation adaptation of suicide safety planning intervention using peer support in rural areas

**DOI:** 10.3389/frhs.2023.1225171

**Published:** 2023-12-22

**Authors:** Eva N. Woodward, Amanda Lunsford, Rae Brown, Douglas Downing, Irenia Ball, Jennifer M. Gan-Kemp, Anthony Smith, Olympia Atkinson, Thomas Graham

**Affiliations:** ^1^ Department of Psychiatry, University of Arkansas for Medical Sciences, Little Rock, AR, United States; ^2^ VA Center for Mental Healthcare & Outcomes Research, Central Arkansas Veterans Healthcare System, Little Rock, AR, United States; ^3^ Arkansas Freedom Fund, Little Rock, AR, United States; ^4^ Department of Medical Humanities and Bioethics, Center for Health Literacy, University of Arkansas for Medical Sciences, Little Rock, AR, United States; ^5^ College of Medicine, University of Arkansas for Medical Sciences, Little Rock, AR, United States

**Keywords:** patient and public involvement, community engagement, community-based participatory research, implementation science, suicide prevention, adaptation

## Abstract

**Introduction:**

Currently, seventeen veterans die by suicide daily in the United States (U.S.). There are disparities in suicide behavior and access to preventative treatment. One disparity is the suicide rate in rural areas, including the state of Arkansas—suicide deaths among rural veterans increased 48% in the last 2 decades, double that of urban veterans. One major challenge for veterans in rural areas is the lack of healthcare providers to provide Safety Planning Intervention, which is an effective intervention to reduce suicide attempts in the general adult population and among veterans. One solution is more broadly implementing Safety Planning Intervention, by using peers to deliver the intervention in rural communities. Before implementation, the intervention needs to be adapted for peer-to-peer delivery, and barriers and facilitators identified.

**Methods:**

Since January 2021, using community-based participatory research, we collaboratively developed and executed a 1 year study to adapt Safety Planning Intervention for peer-to-peer delivery in rural communities and identified implementation barriers and facilitators prior to spread. From July 2022 to February 2023, we conducted group interviews with 12 participants: rural veterans with prior suicidal thoughts or attempts in one U.S. state, their support persons, and healthcare professionals with expertise in veteran suicide prevention, Safety Planning Intervention, and/or peer delivery. We collected qualitative data through interviews during nine, 2 h meetings, and quantitative data from one anonymous survey and real-time anonymous voting—all on the topic of core and adaptable components of Safety Planning Intervention and implementation barriers and facilitators for peer delivery in rural communities. Questions about adaptation were designed according to processes in the ENGAGED for CHANGE community-engaged intervention framework and questions about facilitators and barriers were designed according to the Health Equity Implementation Framework. Participants categorized which Safety Planning Intervention components were core or adaptable, and how freely they could be adapted, using the metaphor of a traffic light in red (do not change), yellow (change with caution), and green (change freely) categories.

**Results:**

Participants made few actual adaptations (categorized according to the FRAME modification system), but strongly recommended robust training for peers. Participants identified 27 implementation facilitators and 47 barriers, organized using the Health Equity Implementation Framework. Two example facilitators were (1) peer-to-peer safety planning intervention was highly acceptable to rural veterans; and (2) some state counties already had veteran crisis programs that could embed this intervention for spread. Two example barriers were (1) some community organizations that might spread the intervention have been motivated initially, wanting to help right away, yet not able to sustain interventions; and (2) uncertainty about how to reach veterans at moderate suicide risk, as many crisis programs identified them when suicide risk was higher.

**Discussion:**

Our results provide one of the more comprehensive pre-implementation assessments to date for Safety Planning Intervention in any setting, especially for peer delivery (also referred to as task shifting) outside healthcare or clinical settings. One important next step will be mapping these barriers and facilitators to implementation strategies for peer-to-peer delivery. One finding surprised our research team—despite worse societal context in rural communities leading to disproportionate suicide deaths—participants identified several positive facilitators specifically about rural communities that can be leveraged during implementation.

## 1. Introduction

Currently, 17 veterans die by suicide daily in the United States (U.S.) (1). There are disparities in suicide behavior and access to preventative treatment. One disparity is the suicide rate in rural areas, including the state of Arkansas—suicide deaths among rural veterans increased 48% the last 2 decades (2), double that of urban veterans (1). The disparity between rural and urban veterans’ suicide rates may be associated with factors related to contextual societal hardships veterans face in rural areas, such as more chronic poverty, fewer economic investments (3, 4), not enough healthcare services (5, 6), and long distances to travel for healthcare (7). Another reason for delayed care are cultural beliefs about relying on one's self to handle mental health problems (8, 9).

Although there are evidence-based interventions to prevent suicides, such as Safety Planning Intervention (which includes lethal means safety counseling, they are primarily delivered in healthcare settings that can be hard to access for rural veterans due to long drives or limited internet access to use telehealth (10). Safety Planning Intervention is an evidence-based intervention, a one-time interaction in which a healthcare provider collaborates to complete a “safety plan” with a veteran who is experiencing suicidal thoughts but does not require inpatient psychiatric hospitalization (11). In Safety Planning Intervention, the provider learns the patient's most recent suicidal crisis, explains safety planning is a collaborative effort between them, and together, they complete a 6 step safety plan with the veteran to cope with suicidal thoughts until they pass, or clarifying how veteran can seek emergency assistance (12). Among veterans in five emergency departments, Safety Planning Intervention was associated up to 45% reductions in suicidal behavior (13). The last step in a safety plan involves lethal means safety counseling, during which providers and patients discuss ways to restrict access temporarily to means in the patient's environment they may use to attempt suicide (e.g., disassembling firearms, placing pain medicine in a locked box) (12). Lethal means safety counseling is key for veterans (14) as 68.2% of veteran suicides occur by firearm (1). Risk of firearm suicides for rural veterans is even higher than in urban areas, because firearm ownership and availability is greater (15, 16). However, one major challenge for veterans in rural areas is lack of healthcare providers to provide Safety Planning Intervention (6). This is especially true in Arkansas where every county is “medically underserved” without enough providers (17). As part of a public health approach, community engagement in veteran suicide prevention is essential to promote health equity by addressing suicide risk in rural communities (18, 19). One solution is implementing peer-to-peer delivery in rural communities (also referred to as task shifting).

Veterans’ preference is to first have discussion about securing lethal means—items a veteran might use to harm themselves, such as firearms or pain medication—with family members or peers (20). Having peers deliver mental health care in some situations has been as effective as when professionals deliver this care (21). In one study, veteran peers were trained to deliver a brief suicide intervention to other veterans who had been in a psychiatric hospital due to suicide risk (22). Results indicated peers were able to deliver the intervention very close to how it was intended to be delivered, and veterans receiving the intervention reported highly positive experiences discussing suicide prevention with peers (22). Peer support for veteran suicide prevention is so promising that the Veterans Health Administration (VA) has embedded veteran peers in some healthcare clinics and services nationwide (23). Given this, plus the dearth of healthcare coverage in rural areas like Arkansas, our community-academic research partnership between the Arkansas Freedom Fund (veteran community organization), Center for Mental Healthcare and Outcomes Research at the Central Arkansas Veterans Healthcare System, and the University of Arkansas for Medical Sciences decided to culturally adapt and prepare Safety Planning Intervention to be delivered by peers outside healthcare settings, i.e., community members living in rural areas.

## 2. Materials and methods

In the current study, we used scientific methods for community engaged adaptations of interventions, community based participatory research (CBPR), and implementation science, relying on data generated by veterans with lived experience with suicidal thoughts or attempts (community) and healthcare professionals with expertise in suicide prevention. Since January 2021, our community-academic research team has met every 2 weeks to collaboratively develop research questions, study design, and execute the study. From January to September 2021, we collectively participated in a local university course on community-based participatory research designed for community-academic research teams. This course included online modules, live video courses, textbook readings, and team mentoring with an experienced community engagement researcher to assist us in preparing a grant application for this study. To ensure every team member provided input, accommodations were made for amputee veterans, limited internet services, and team members who were not comfortable speaking in a group. By making accommodations to receive input from every team member, we were able to thoroughly understand others’ needs for our research and what study modifications were needed after the study commenced. This study was co-led by an academic principal investigator and a community principal investigator. Our first aim was to determine core and adaptable components of Safety Planning Intervention that could be used in implementation of the intervention peer-to-peer in community organizations. Our second aim was to identify anticipated implementation barriers and facilitators if deploying this in real world settings, to prepare for spread and scale through community organizations.

### 2.1. Design

To guide adaptation, we use the ENGAGED for CHANGE framework (24). This framework presents 13 steps for how to develop an intervention with community members. ENGAGED for CHANGE is an acronym signifying each step using community needs, priorities, and assets; existing data; and relevant theory (24). We aimed to complete the following 8 steps because this was most feasible in our 1 year timeframe and aligned with our aims: (1) Expand the partnership, (2) Intervention team established, (3) Gather existing literature, (4) Assess community needs, priorities, and assets, (5) Generate and refine intervention priorities, (6) Evaluate and incorporate meaningful theory, (7) Design an intervention logic model, and (8) Create objectives, activities, and materials. Because Safety Planning Intervention already existed, we modified steps of the framework slightly to culturally adapt (instead of develop) Safety Planning Intervention from being delivered by healthcare professionals to being delivered by peers in rural communities.

We used mixed methods research, QUAL + QUAN design, in which qualitative and quantitative data were collected concurrently throughout the study and priority was equally shared between both data types (more detail in Analysis section) (25). Qualitative data were collected each workgroup meeting by asking participants open-ended or “fill in the blank, then elaborate” questions. Quantitative data were collected first through a one-time anonymous survey to participants about their ratings on whether each Safety Planning Intervention step should be retained or change, and if changes, their preferences on how. These data were used to narrow the qualitative questions for the group to steps in which there was not quantitative consensus in the survey, and to make decisions about adaptation about steps in which there was quantitative consensus in the survey. After using qualitative questions and data during workgroup meetings, we then refined specific a quantitative question about each proposed adaptation, asking participants to rank their agreement with the adaptation in real-time voting during workgroup meetings.

### 2.2. Participants and recruitment

We convened research study participants known as the “Arkansas Safety Planning Intervention Workgroup” at nine monthly meetings. Participants included: rural Arkansas veterans with prior experience with suicidal thoughts and/or attempts, support persons of those veterans, rural Arkansas veterans who worked in community organizations to prevent veteran suicide but did not necessarily experience suicide thoughts or attempts themselves, mental health and internal medicine professionals with expertise in suicide prevention and/or peer support interventions (some working in VA). The goal of the participant workgroup was to decide on core and adaptable components of Safety Planning Intervention, determine adaptations to Safety Planning Intervention for peer-to-peer delivery, and identify preliminary implementation barriers and facilitators. The Arkansas Safety Planning Intervention Workgroup followed the ENGAGED for CHANGE steps, digesting data from different sources of knowledge, including veterans’ lived experience, government reports, existing training manuals and tools for Safety Planning Intervention, and published peer-reviewed research. This group was distinct from our research team, the latter of which oversaw and coordinated the study with community partners embedded as a co-principal investigator and significant contributors. Both groups had members from the community with lived experience.

Regarding eligibility criteria, we recruited veterans from rural communities to become participants and members of our Arkansas Safety Planning Intervention Workgroup. To be eligible to participate, veterans had to meet these inclusion criteria: they must have formerly served in the U.S. military, had suicidal thoughts or attempts before but not within 6 months, and live in a rural community with a Rural Urban Commuting Area code of four or higher (higher codes equal more rural places) (26). We also allowed people who were identified by these veterans as close family, friends, or peers, who were support persons to the veteran participant through a suicidal crisis. For recruiting healthcare professionals, our inclusion criteria were centered around their expertise in relevant topics: either suicide prevention, veteran mental health, and/or peer support.

To recruit veterans and their support persons participants, each community-academic research team member sent an initial outreach to potential participants via text message, e-mail, and social media pages of their own and of the community organization partner using scripted text with a flyer about the purpose of the study, roles for participants, risks, benefits, compensation, and contact information. Forty-one people were interested. From this initial pool, 19 people were not responsive to follow-up for screening, and 22 potential participants agreed to complete eligibility screening questions by any member of the community-academic research team. During eligibility calls, two individuals’ phone numbers were not working, 5 were not eligible due to not living in a rural area, three declined to participate [did not want to commit to monthly meetings (*n* = 2), was not interested (*n* = 1)], one person was hospitalized and later died, and two were leaders of other community groups that agreed to forward study information. We recruited 12 total participants: 6 veterans, 3 support persons, and 3 healthcare professionals. All participants completed at least two meetings and 91.7% completed the anonymous survey. One support person only attended two of the workgroup meetings and completed the survey and one of the healthcare professionals only attended one meeting (reasons unknown), and the remaining ten participants engaged in majority of meetings. See Table 1 for description of the initial sample.

**Table 1:** Demographic characteristics of veteran and support person participants.

Demographic characteristic	*N* (%)
Age	Mean = 49.78 years, Range = 33–62 years
Veteran status	
Veteran	6 (66.67%)
Not a veteran (support person)	3 (33.33%)
Gender identity	
Man	4 (44.44%)
Woman	5 (55.56%)
Racial identity	
American Indian or Alaska Native	1 (11.11%)
White	7 (77.78%)
Declined to report	1 (11.11%)
Ethnicity	
Hispanic or Latino	1 (11.11%)
Not Hispanic or Latino	8 (88.89%)
Sexual identity	
Straight or heterosexual	7 (77.78%)
Lesbian, gay, or queer	2 (22.22%)

### 2.3. Procedures, data collection, and analysis

#### 2.3.1. Procedures

We assembled the Arkansas Safety Planning Intervention Workgroup for nine, 2 h meetings from July 2022 to February 2023, approximately every 3–4 weeks. The first and last meeting were hosted in-person in the public library, rather than our hospital clinics, in a convenient central city agreed upon by all participants. The purpose of in-person meetings was relationship building and had a hybrid video conference option for those who could not attend in person. The remaining seven meetings were hosted via video/telephone conference as this was preferred by participants and the research team due to participants being geographically dispersed and ongoing COVID-19 infection concerns.

To enhance equitable participation and minimize power imbalances, we started with only community member participants at the first three meetings and met with the healthcare professional participants in one meeting separately, so all could become oriented to the topic and process with people more like themselves (Table 2). We incorporated the healthcare professional participants into combined meetings with community participants after all community participants reported they were ready for that shift. We allowed participants to make decisions about whether they wanted their video camera on or off, and to respond verbally or type in the chat text box. We ensured each participant's name on the video conference reflected what they wished to be called. We alternated the meeting time each month from during daytime to evening hours to accommodate different schedules.

**Table 2:** Meeting numbers, topics, and participants aligned to ENGAGED for CHANGE framework steps to adapt an existing intervention and identify preliminary implementation barriers and facilitators.

Meeting topic and data collection by ENGAGED for CHANGE step	# of meetings on topic and participants
**E**xpand the partnership	Prior to study
i**N**tervention team established	Prior to study
Bonus: created community agreements about ground rules for discussion **G**ather existing literature • Community: review written summaries of safety planning intervention and data on suicide risk factors for rural veterans; bring their expertise from other sources to blend with research expertise (interview) • Professionals: review safety planning intervention steps and determine what they perceive as core/adaptable functions and forms of the intervention (interview)	1 (community); 1 (professionals)
**A**ssess community needs, priorities, assets • Open-ended questions about anticipated implementation barriers and facilitators for future spread (interview)	1 (community) 1 (combined)
**G**enerate and refine intervention priorities • Create draft of core functions and forms of the intervention to begin to focus intervention goals on community needs and priorities (interview + survey + real-time voting)	2 (combined)
**E**valuate and incorporate meaningful theory • Identify and incorporate any theories that may provide systematic knowledge check of existing perspectives (interview) • Refine list of red/yellow/green light adaptations (interview + survey + real-time voting)	1 (combined)
**D**esign an intervention logic model • Draft and finalize a visual logic model to depict links between suicide risk factors, intervention functions and forms, and hopeful outcomes (interview)	1 (combined)
**C**reate objectives, activities, materials • Determine which materials and activities need to be adapted (interview + real-time voting) • Generate list of important measures of acceptability, feasibility, and fidelity for future pilot (interview + real-time voting)
	1 (combined)
Bonus topic: “member checking” results including (1) Red/Yellow/Green light adaptations, (2) implementation barriers and facilitators, (3) logic model with measures for future pilot to finalize analysis and interpretation (interview + real-time voting)	1 (combined)

##### 2.3.1.1. Positionality of qualitative interviewers

One academic member *and* one community member from the research team co-facilitated workgroup meetings (functioning as qualitative interviewers in the group setting). The academic member (blinded for peer review) was a PhD clinical psychologist and lead researcher for the study. She identified as a white cisgender woman who had not served in the U.S. military, and had provided mental healthcare to veterans for 11 years through VA. She made connections with the 3 healthcare professional participants through professional networks. She met 2 participants from the veteran community and one support person participant prior to the study during community volunteering. This was this interviewer's first experience leading community engaged research, and her prior training on the topic included one doctoral-level course, monthly mentoring for 2 years, and the community-based participatory research course with the community organization partner mentioned in the introduction. She believed community engaged research and Safety Planning Intervention were valuable, although not sufficient, to reduce veteran suicides. She grew up in a rural community and understood many socioeconomic challenges reported by participants. The community member (blinded for peer review) was a veteran. He identified as a white male that grew up in urban and rural settings. He received his Master of Organizational Leadership degree in 2021 and retired as a Senior Master Sergeant (paygrade E-8) in 2022. He served 25 years in the Air Force with 12 years as a Senior Noncommissioned Officer and 6 years as a First Sergeant. This position is responsible for quality of life for all service members and their families, which gave him innumerable experiences with mental health and suicide situations. He responded to and guided people through their own suicidal ideation and was himself diagnosed with depression in 2021 when he noticed himself developing his own plan for attempting suicide. He was motivated for the study because he saw first-hand the impact a single person can have on another person's life just by responding to them in their time of need. He knew some of the research participants before the study from a veteran community organization. To assist the research participants, he shared his personal story and conducted role plays during meetings with the research lead to show examples of Safety Planning Intervention. Another research team member (blinded for peer review) was present at all meetings to take notes and answer questions regarding logistics such as scheduling and participant payment; they did not conduct group interviews.

##### 2.3.1.2. Group meeting processes

The process used for each workgroup meeting followed a general agenda of greeting each other, reviewing community agreements made collaboratively as ground rules for discussion, presenting information needed to discuss the topic, open-ended or “fill-in-the-blank and elaborate” questions about the topic, and if related to making decisions about adaptations, anonymous real-time voting on adaptations. Tasks varied each meeting depending on the step from the ENGAGED for CHANGE framework that allowed us to meet research aims; see Table 2 for a list. We also added one meeting for member checking, a method in qualitative research to enhance internal validity of results (27). For member checking in our study, we analyzed results and presented them to research participants in writing 1 week before meeting, and discussed them verbally in a 2 h meeting, asking questions to expand depth, clarify errors, or add missing data.

#### 2.3.2. Data collection

##### 2.3.2.1. Qualitative interviews

The primary data collection method was group qualitative interviews, documented through audio recording and intensive notetaking during meetings to identify: (1) adaptations; (2) potential barriers and facilitators to eventual implementation and spread of the adapted intervention; and (3) creating a community-academic logic model of how peer-to-peer Safety Planning Intervention might reduce suicide risk (not the focus of this manuscript). Qualitative questions about adaptations were broader in initial meetings and became more focused closer to making decisions. Questions were designed before each meeting by the two meeting co-facilitators. Before participants completed the anonymous survey about adaptation preferences and their priority (i.e., red/yellow/green light), broad question examples were “What parts could remain the same and would work if a peer were doing this with a veteran?,” What parts do you think should be changed and why?” and “What do you think is the least/most important aspect? Why?” After reviewing the survey (below), co-facilitators created narrow qualitative questions about each step for discussion, asking specifically for thoughts on each step of the safety planning process in which there was not consensus.

##### 2.3.2.2. Survey

After three meetings using qualitative interviews with workgroup participants, our research team realized we might not be hearing every participant's voice when it came to making decisions about adaptations to Safety Planning Intervention peer-to-peer. Suggested by community members on our research team, we modified our protocol and created a one-time anonymous survey about each step in the Safety Planning Intervention process as traditionally used in healthcare settings. We sent participants the survey through a web-based survey platform, RedCAP, and ensured it was anonymous by not tracking their IP address nor asking for identifiable information. Questions asked about comfort sharing during group meetings and also asked participants to review each step of safety planning and whether they wanted to retain that step, change it, or were unsure about adaptation (see Supplementary File).

If there was clear consensus with >70% of participants reporting the same preference for the adaptation on the survey, the researcher team recorded this as a decision, still presenting it at the next meeting to workgroup participants as a decision on which they predominantly agreed. When there was not clear consensus on an adaptation in survey results, data were used to formulate qualitative questions at the next two meetings to clarify perspectives, elaborate on differing viewpoints, generate possible adaptations, and eventually, narrow options for voting (see below).

##### 2.3.2.3. Real-time voting

For each adaptation decision made, after discussion and some verbal indication of consensus in workgroup meetings (not anonymous), group interviewers created a voting poll using Zoom videoconference software in real-time to assess whether participants agreed or disagreed with a proposed decision for adaptation. All participants were requested to vote anonymously on the poll during the meeting whether they (1) “mostly agreed” or “strongly agreed,” (2) were “ unsure,” or (3) “mostly disagreed” or “strongly disagreed” about the adaptation. Decisions were considered final when all participants anonymously voted they “mostly agreed” or “strongly agreed.” If anyone voted for other responses, discussion continued until a decision was made.

#### 2.3.3. Data analysis

Data were integrated after each data collection point in an ongoing, iterative template analysis and interpretation process (28, 29). Using audio recordings and summary meeting notes (not transcription), two research team members served as coders of qualitative data in between each meeting and record quantitative data results as well (blinded for peer review), which would then be brought to the entire research team where they would interpret findings and use them to inform the next meeting topic. One coder was the research PI and the other was a research assistant who was present at all workgroup meetings and received training in qualitative template analysis from the PI for this study.

For all analysis, we sorted qualitative and quantitative data into templates, reflecting data gathered at one point in data collection. The templates were organized into three categories aligned to research aims: (1) adaptation suggestions or decisions, (2) implementation barriers and facilitators, or (3) other (which over time, became training for peers in Safety Planning Intervention, informed by qualitative data) (see Supplementary Files). For adaptation, we merged quantitative findings from the one-time survey and real-time voting with qualitative data about preferences. For implementation barriers and facilitators, we used only qualitative analysis. Coders reviewed meeting notes and audio recordings and, if relevant, survey findings or real-time voting results, and create separate templates independently. Then, they would meet to compare findings in their templates, discussing divergence, referring to original data as needed, and ultimately, creating one master template of each meeting. Together, the coders would extract data from the master template into one of three formats that served as “deliverables” for this study and allowed for clearer interpretation:

1. FRAME system to track adaptations made to Safety Planning Intervention (30),
2. Red/yellow/green traffic light categories for categorizing how freely a peer could adapt each step of Safety Planning Intervention (31), and
3. Barriers and facilitators to implementation of peer-to-peer Safety Planning Intervention organized using domains of the Health Equity Implementation Framework (32)

##### 2.3.3.1. Analytic frameworks

The FRAME coding system enables teams to note multiple facets of adaptations to an intervention, including but not limited to when it was made, why, type of modification, and who prompted the adaptation. Red/yellow/green traffic light categories have been used as a dissemination tool in training people learning an intervention on what should be modified and what should remain intact. Using the metaphor of a traffic light in the U.S., each color of the traffic light signifies what intervention components are core and should be retained in their original state (red light), could be adapted but should occur with caution or under certain conditions (yellow light), and can be adapted freely (green light). The Health Equity Implementation Framework is an implementation science framework documenting factors of successful and *equitable* implementation. Applied to this study, domains include factors about Safety Planning Intervention peer-to-peer, peers delivering the intervention, veterans in need of a suicide safety plan who are at moderate risk of suicide, interaction between a peer and a veteran to engage in Safety Planning Intervention, local contexts within community organizations, the U.S. state, and broader societal contexts including VA healthcare, sociopolitical forces such as laws or policies, economic concerns such as what goods might be exchanged for safety planning, and physical structures such as the built environment in rural communities where safety planning might occur (33).

Because the coders had analyzed data in between each meeting, they were prepared to present preliminary results to workgroup participants at the last meeting for member checking (see Table 2). Coders incorporated all additions and elaborations recommended by participants from that meeting, although they were minor. Final analyses were presented to the entire research team, who assisted with interpretation and finalized results.

## 3. Results

### 3.1.1. Adaptations to safety planning intervention for peer-to-peer delivery

One major finding from this study was that participants declined to significantly change the intervention, but strongly recommended robust training for peers and had suggestions about content for the training (the latter not the focus of this manuscript). Although there appeared to be diverse opinions initially on quantitative survey ratings, once those topics were discussed with participants, they asked questions, heard others’ viewpoints, and learned more about Safety Planning Intervention, and ended with unanimous real-time voting results on every decision to adapt (or not) Safety Planning Intervention peer-to-peer. They made few actual adaptations.

The adaptations to Safety Planning Intervention from professionals delivering it in healthcare settings to peers delivering it in community settings included: (1) the intervention can be delivered by peers in the community; (2) coping strategies and social settings for distraction must fit the veteran's life situation including housing, social connections, physical ability, interests, transportation, and income; (3) peers providing safety planning can have written or pictorial information such as visual aids showing the efficacy of safety planning and the suicide risk curve; (4) when peers are learning about the veteran's suicidal crisis, this information may be in the form of written thoughts from the veteran, a text message, received face-to-face, or via telephone; and (5) an optional follow up contact line can be added to the bottom of the safety plan copy if there is a safe and clear choice for follow up. See more modification details in FRAME coding log as a Supplementary File.

Participants also categorized each step in the process of conducting a safety plan as core or adaptable using the red/yellow/green traffic light categories (see Table 3). As an example of a core component in the red light category, participants reported the first 3 steps in the process of safety planning (not the first 3 steps on a written safety plan) should occur if a veteran at moderate suicide risk was willing and able, and otherwise, they should not proceed with safety planning. Those first three steps are: (1) get the veteran's story of most recent suicidal crisis, (2) show veterans the suicide risk curve, and (3) explain suicidal thoughts come and go. This is an example of an instruction in how to conduct safety planning trained to healthcare professionals that the workgroup participants in our study chose to retain. An example of an adaptable component that should only be adapted with caution or under certain conditions was about peers asking a veteran with whom they would like to share the safety plan, if anyone. Participants agreed that peers could ask this if the veteran had social contacts listed on the plan but should not ask if the veteran could not identify anyone to call for distraction or crisis in earlier safety planning steps. An example of an adaptable component that could be freely altered was how the peer received the veteran's recent suicidal crisis story—it could be received in any format including written, text message, verbal in person, or verbal via telephone call.

**Table 3:** Core and adaptable parts of safety planning intervention for peer-to-peer delivery by red, yellow, and green traffic light categories.

Red light—must not change (core)
• The first three steps of safety planning should happen if veteran is willing and can do the safety plan:
○ Get the veteran's story of most recent suicidal crisis
○ Show veteran the suicide risk curve
○ Explain suicidal thoughts come and go
• Safety planning should not be done with people who are intoxicated, high, or cannot problem solve to complete safety planning
• When understanding veteran's most recent suicidal crisis, the peer needs to understand what got the veteran to that point, so the peer may need to ask follow-up questions.
• The peer must tell veterans the safety planning process is a team effort.
• Listing warning signs should remain as first formal written step before working on other steps of the plan
• Peer must ask veterans if their personal warning signs listed would signal a crisis is coming or remind them to use the safety plan
• For the following steps, they must fit the veteran's life situation including housing, social connections, physical ability, interests, transportation, and income:
○ List *distractions* they can use by themselves (internal coping strategies)
○ List people to call or places to go for *distraction*
○ List at least one person to call for help with *crisis*, including professionals, regional sources of healthcare, hotlines, or a peer
• The peer must explain how to follow the safety plan (e.g., as soon as they feel better, they can stop, also can skip to whatever step they want)
• The peer must ask about the chances of veteran actually using safety plan. Examples include: How likely are you to use this? How comfortable are you in using this properly? Do you see any barriers to using the safety plan?
• Veterans must get a copy of their safety plan
Yellow light—can make changes with caution (adaptable)
• Peers can also have written handouts on some facts about need for safety planning and other pictures like the risk curve, but do not need this.
• The following steps are very important and ideally should be done in order, but if veteran is having difficulty, these steps could be skipped and come back to later, or left empty if unable to brainstorm good ideas for safety plan:
○ List *distractions* they can use by themselves (internal coping strategies)
○ List people to call or places to go for *distraction*
• Lethal means safety counseling should happen at the end of safety planning where it is listed but could happen at any step before conversation ends if there is limited time, an interruption, or needs to end before the entire plan is completed.
• When discussing lethal means, another support person can be discussed or brought in the conversation but does not have to be.
• It is important to make sure veterans have a clear place they will access the safety plan, but the peer should be aware it may be redundant depending on format.
• Peer can ask veteran who they would like to share the safety plan with depending on whether the person had social contacts listed on the plan or not. Do not ask if they could not identify anyone to call for distraction or crisis.
• Adding a line on the safety plan for follow up contact can happen if there is a safe and clear choice, but is not required.
Green light—changes can be freely made (adaptable)
• How the peer explains the suicide risk curve to veterans can be in any format—draw out it, show a written handout, or use hands to show, while explaining verbally
• Veteran's recent suicidal crisis story can be received in any format including written, text message, verbal in person, or verbal via telephone call.
• Safety plan can be in any format such as photo, screenshot, smartphone app, wallet size card, or full-size paper.

### 3.1.2. Barriers and facilitators to implementing peer-to-peer safety planning intervention

Regarding implementation barriers and facilitators, participants identified 27 facilitators, or strengths to harness, and 47 barriers, or challenges to overcome or plan to work around, should peer-to-peer Safety Planning Intervention be deployed in community settings. All were categorized into domains of the Health Equity Implementation Framework (see Table 4). Among facilitators, majority were identified in domains of the intervention itself, peers who would be offering safety planning, and factors in rural Arkansas and our existing healthcare systems. For example, regarding the intervention itself, participants perceived it very favorably with relative advantage over current suicide prevention interventions participants experienced (e.g., some advantages to medication because it gave veterans tools to use). Among peers who would offer safety planning, one major strength participants reported was peers with suicide experience were very motivated to help, as one community participant said, “*I want to make sure that, after all the effort my family put into saving me, it’s not lost on the next generation.*” (Veteran, age 55, identified as male) Among the state-level context, specifically our healthcare systems and rural communities, participants identified one strength being multiple existing generations of veterans here in Arkansas to help each other. See Table 4 for a list of implementation facilitators.

**Table 4:** Implementation barriers and facilitators to spreading peer-to-peer safety planning intervention in rural Arkansas community organizations.

About safety planning intervention veteran-to-veteran in rural Arkansas
Facilitators
• Easier for veterans to relate to other veterans—peer delivery is very acceptable.
• Peers can share their experiences with the veteran in crisis, which may lead to trust and a greater connection.
• Veterans feel safety planning is important and has potential to be impactful.
• Safety planning is something to learn with skills to use—a relative advantage over current management of higher suicide risk (e.g., medications).
• Peer-to-peer safety planning is important because most providers are not trained in safety planning and a veteran peer can make a bond with another veteran that some providers cannot make.
Barriers
• Some veterans will not tell the whole truth to questions that are sensitive or personal.
• The safety planning process may take a while and the veteran may walk away or disengage.
Recipients: about veterans considering suicide—at moderate risk in rural areas
Facilitators
• For people who are religious, trusting in a higher power and having faith is a resource (culturally relevant belief).
• Safety planning, especially steps about connections with others, plant a seed that others value them and enhance the veteran's self-worth.
• Safety planning is a good start to find out what works and what does not, possibly bridge to treatment, and is flexible for the veteran.
Barriers
• People who are actively using drugs or alcohol might not be able to engage in a safety plan.
• Some veterans have distrust in the government, although this is lessened by peer outreach (culturally relevant belief).
• Some veterans are programmed from military training not to discuss problems, to keep secrets (culturally relevant belief).
• Veterans do not discuss mental health enough.
• Veterans trained to be combative in military, and if frustrated, might be combative with a peer trying to help (culturally relevant belief).
• Veterans with personal suicide experiences are a minority even though it is a huge problem. It may be hard to find these Veterans in crisis.
• Veterans are fearful they will be “locked up” and cannot get out, so delay seeking mental healthcare (culturally relevant belief).
• Veterans may not be able to think of a list of contacts or healthy distractions, it may take a while to come up with answers.
• Veterans may fear others will take away their firearms or important medications.
• Feeling of being a burden to others prevents calling on others during times of crisis.
Recipients: about peers offering safety planning (not trained professionals)
Facilitators
• Peers are unrelated to any institution—not medical, not police, not government—and so might be more acceptable.
• Peers have “*walked in their shoes*” (connection), might have a safety plan themselves, and speak from experience about usefulness. (V1, age 62, identified as male)
• Peers with suicide experience are very motivated to help. “*I want to make sure that, after all the effort my family put into saving me, it’s not lost on the next generation*.” (Veteran, age 55, identified as male)
• People in the military work with people across difference—race, state of origin, sexual identity, and can stay focused on “*finishing the mission*.” (Veteran, age 62, identified as male)
Barriers
• May not be enough peers to match veterans based on lived experience (e.g., women sexual assault survivors, suicide survivors).
• Peers will be exposed to scenarios that are triggering to them based on their lived experience (e.g., active drug use).
• Safety concerns for peers: veterans or their families could be potentially dangerous to peers.
• Peers will need scripts, checklists, and a lot of training and debriefing to feel comfortable doing safety planning.
• It will be hard to figure out how to assign a certain number of veterans per peer so not overloading one peer.
• Unclear how peer support will be different from clinicians.
• Without written notes documented and stored by peers, it would be hard for one peer to pick up where another peer left off.
• Where would peers store safety plan with veteran's information securely if they use it for follow up?
• Peers fearful to call veterans to follow-up in the event the veteran is in immediate crisis and peer does not know what to do.
Interaction: the moment when a peer tries to connect with a veteran considering suicide
Facilitators
• Timely—being able to offer it when they need it, rather than wait for a healthcare appointment.
Barrier
• Peers need to establish trust quickly with veterans they do not know. “*You have to prove that you are there to help and not manipulate*.” (Veteran, age 57, identified as male)
Inner context: state-level community and professional settings where safety planning intervention might be delivered, including organizations in Arkansas (e.g., VFW, American legion, churches)
Facilitators
• Multiple veteran groups are available to spread safety planning as many have established networks statewide. They can also train peers.
• Some gun manufacturers and VA give gun locks and medication storage as a resource.
• One good entry into each community is the county Veteran Service Officer through state-level veteran affairs office.
Barriers
• Peers could get calls about someone who is not a veteran (e.g., a veteran's child).
• Training peers in safety planning would need to be regular, with ongoing support so they can deliver it well.
• Some organizations are motivated the moment you talk with them and want to help right away. When actual work is ready, there can be low or no response.
• Professional resources like counselors or doctors are not always available immediately because of other patients and not usually free during evening hours, so peers and veterans would need to have some resources like this available 24/7.
Outer context: state-level settings relevant to peer-to-peer delivery of safety planning intervention in community organizations (including rural areas and healthcare settings in Arkansas)
Facilitators
• Some counties already have veteran crisis programs that we can collaborate with.
• There exist multiple generations of veterans here in Arkansas to help each other.
• We have a state suicide hotline that is not VA (∼5% of veterans who call suicide crisis line choose the state version).
• There are already places who have trained peers and we can learn from them.
Barriers
• May not be enough providers to include as professional contacts on safety plan in rural areas.
• May not be enough police force for safety/wellness checks in rural areas.
• Sometimes in healthcare, providers and staff are rotating so frequently that it is hard to get the same provider twice.
• If emergency department is needed in rural areas, they are often not equipped to assist with suicide prevention.
• Non-VA emergency services or mental health providers need to understand military culture working with veterans (e.g., screening for common problems like post-traumatic stress disorder or traumatic brain injury).
• Current emergency department way of doing things can be further alienating, isolating, and cause anxiety.
• Ambulances take people to the closest emergency department, so they are not guaranteed to go to the VA.
• Not sure where we will find moderate risk veterans, as existing veteran crisis programs tend to serve people at imminent risk.
• Moving through healthcare systems is hard, and can be a taxing job for caregivers or families to help veteran.
Broader VA-related contexts
Facilitators
• VA mental healthcare is available to any veteran who presents or calls. They can see a provider in person or via video that day in primary care or the mental health clinic and the veteran does not have to be enrolled at the VA.
• There are different types of suicide prevention treatments available in VA and all of them are evidence-based.
• There is an entire psychotherapy program for suicide prevention available at the regional level.
• Providers have experience dealing with veterans and veterans have different issues than the civilian population.
Barriers
• Rides to VA healthcare offered by Disabled American Veterans are not accessible—too early, run only once daily in the morning.
• There is a belief that some veterans aggravated the VA healthcare system and got banned so are unable to use those services.
• Some veterans have bad feelings towards VA based on negative experiences such as “*they don’t help and they don’t care*” so it is hard to use VA as a professional resource reliably. (Veteran, age 33, identified as female)
Economic or financial factors
Facilitators
There are a lot of free, trustworthy online resources for managing mental health distress to apply at home (e.g., apps, youtube channels, websites such as Urban Valor, PTSD Coach, Virtual Hope toolbox).
Barriers
• If veteran is not connected to VA, do not have easy or affordable services to refer to (on the safety plan).
• Poverty and economic distress in rural Arkansas led to veterans not having insurance or funds to get care that would be part of a safety plan.
Physical environment in which peers will be conducting safety plans with veterans
Facilitators
• Being outside and away from people can be soothing, calming, and private for sensitive conversations about suicide.
• Many veterans are overwhelmed by crowded places, so fewer people in rural areas is comforting.
• Peer-to-peer delivery is flexible and can go to where the veteran is located. It can even take place on private chats or via telephone in the format the veteran in crisis prefers.
Barriers
• Hard to find veterans in rural areas who are considering suicide, especially those living in secluded environments.
Sociopolitical forces: social norms of our culture, state or county politics, policies, laws or legal factors
Facilitators
• Arkansas has a perceived culture of support for veterans.
• Veterans want to help and support each other, and it lends itself to peer-to-peer safety planning.
Barriers
• Participants heard of situations where police departments took firearms or veterans gave them to police, but firearms were not returned.
• Some stigmas about mental health and drug use problems—e.g., even going to VA community based outpatient clinics in rural areas evokes a belief if you are there, you are there because of drug use or mental health treatment.
• Peer concern about legal liability in making suggestions about suicide prevention, firearm, or medication safety.
• Strong culture of hunting and owning firearms in Arkansas, so if peers are going to bring up gun safety, they need to be careful on wording.
• Structure of the military is not present in veteran status, so the transition from military to veteran status can make it such that veterans are unsure how and when to get help for others.

Regarding implementation barriers, majority of barriers were identified among similar domains in which majority of facilitators were identified: veterans considering suicide at moderate risk in rural areas, peers offering safety planning, state-level contexts including our healthcare systems and rural communities, and sociopolitical forces including social norms, state or county policies, laws and legal factors. Among veterans who might need safety planning, participants reported multiple barriers that would result in a veteran declining or prematurely dropping out of the intervention, even if a one-time interaction, including culturally relevant beliefs that veterans are programmed from military training not to discuss problems, especially mental health problems. Among peers offering the intervention, participants reported several implementation barriers related to logistics and workflow, such as being unsure if or how peers should document Safety Planning Intervention like healthcare professionals do and where they would store that information. In the state-level context domain, one barrier reported was that they reported if a peer should need to help a veteran access an emergency department in a rural area, emergency departments were not perceived as equipped to assist adequately with suicide prevention. Among the sociopolitical domain, one barrier for implementing this intervention, which involves discussion of safe firearm and medicine storage when in suicidal crisis, is that there was a strong culture of hunting and owning firearms in Arkansas, so if peers are going to bring up gun safety, they need to be careful on wording so as not to cause defensiveness among veterans. See Table 4 for full list of implementation barriers.

## 4. Discussion

In this study, we assembled a workgroup of participants in one U.S. state, Arkansas, to adapt Safety Planning Intervention from being delivered by healthcare professionals in clinical settings to being delivered by peers without medical or mental health training in community settings. Through multiple group interviews, a one-time anonymous survey, and anonymous real-time voting during workgroup meetings, participants suggested few changes to adapt Safety Planning Intervention delivered peer-to-peer. Initially, in survey results, there appeared to be much difference in opinion on whether intervention components were core or adaptable, and after discussing these items with open-ended questions and role plays of what safety planning looks like traditionally delivered by a healthcare professional, participants formed consensus on adaptations with few changes to the intervention delivery or format. One concern might have been that the consensus resulted from social desirability bias or perceived pressure to conform, however, we used several strategies to offset this and believe research participants genuinely agreed on adaptations over time. One strategy we used was to closely review and record each participants’ anonymous survey results because the quantitative ratings made it appear their views were quite divergent from one another, yet their qualitative responses in the open-text boxes showcased more of a cohesive viewpoint. Another strategy we used was to ensure anonymous ways of decision making with a range of response options (rather than only agree/disagree)—the survey was anonymous, and at the end of discussing each possible adaptation, we created anonymous polls in real-time to ask how much participants agreed or disagreed with the proposed change. Our final strategy was to ensure the group interviewers were not espousing any position on each component of the intervention, rather, they made efforts to clarify participants’ viewpoints and roleplay examples to showcase an intervention step.

One concern could be whether having community members suggest core and adaptable components is recommended. Similar to other work to merge community and professional knowledge on adaptation (34), we included healthcare professionals with content expertise, meeting with them separately from and together with community members, such that their perspectives informed decision making. Also, involving community members with lived experience in intervention adaptation is essential to ensuring the intervention is useful, supported by an entire field (human-centered design) dedicated to ensuring interventions are tightly mapped to needs of the end-user to optimize implementation (35). Prior research found community input into implementation to result in better intervention fidelity (36), better health outcomes (37), and reduced inequities in access to, satisfaction with, and quality of care (38).

Overall, community members reported Safety Planning Intervention was acceptable and desirable (see Table 4), which matches another study finding high feasibility and acceptability among professionals in healthcare clinical settings (39). One major recommendation from participants was that peers need intensive training with continued supervision and debriefing if providing this service in their communities. Indeed, higher *quality* of Safety Planning Intervention has been associated with better patient outcomes, strengthening the need for continued support of peers providing the intervention (40). In brief (as it is not the focus of this manuscript), community members reported feeling motivated and prepared to assist other veterans with suicide risk and also concerned about not having enough training and potentially harmful effects on peers delivering it, such as the psychological impact of a suicide among their “clients” or even legal ramifications of such situations (among barriers in Table 4). These needs have been recognized by ongoing efforts to properly support peers through ongoing training, supervision, and dissemination of suicide prevention interventions (22, 23), including Safety Planning Intervention using peer-to-peer delivery by one of the developers, the late Dr. Barbara Stanley (41).

Our findings of barriers and facilitators to the implementation of peer-to-peer Safety Planning Intervention showcase a wide range of promising factors to be harnessed and key challenges to plan for in future deployment of the intervention, notably in rural community settings where suicide deaths are higher. One finding surprised our research team—despite societal challenges in rural communities leading to disproportionate suicide deaths in the U.S (3, 4, 8).—participants identified several strengths about implementing this intervention in rural communities. As examples, being in rural settings with fewer people can be less overwhelming for people in suicidal crisis and being outside can be a soothing complement to any suicide prevention intervention. Rural culture can involve a perceived sense of support for one another, which lends itself to offering help during a suicidal crisis. One important next step will be mapping these barriers and facilitators to implementation strategies for this context. Our results mirror those of implementation assessments deploying *other* suicide prevention interventions for rural veterans. Similar to our results, another community engaged initiative to reduce rural veteran suicide also reported the major barriers to uptake of effective suicide prevention interventions were: stigma to seeking mental health care or assistance, fears that having a mental health diagnosis would lead to losing one's right to own firearms, not enough healthcare professionals or clinic environments, including 24/7 crisis centers and psychiatric hospitals, that could adequately assess and treat suicide risk (42). They also reported that one facilitator to implementation is that that community organizations and partners want to be involved quickly and with action-oriented responses (42).

Aside from our study, there are few published reports on assessing implementation context or preparing implementation strategies for spread of Safety Planning Intervention. There is one study suggesting that, in pediatric healthcare settings, collaboration with multiple personnel and brief provider training would be two suggested implementation strategies (43). Another study documented staff perspectives on Safety Planning Intervention in emergency departments (44), finding that the intervention not only helped veterans connect to follow-up services, but also benefitted staff as it increased their comfort in perceiving greater safety for veterans with suicide risk upon discharge. It is possible these findings might translate in peer-to-peer delivery in the community, although this would require further study. The dearth of implementation research on Safety Planning Intervention might be because current implementation strategies are undocumented, naturally occurring in real-world clinical settings (45). A practical next research step would be evaluating the implementation of peer-to-peer Safety Planning Intervention in U.S. rural communities.

### 4.1. Limitations

There are some items which could limit the generalizability of these results. Since data were collected from participants that live in rural areas of Arkansas, these results may not be generalizable to urban or other rural areas across the U.S. The Arkansas Safety Planning Intervention Workgroup included 12 participants, with a predominantly white sample, limiting validity of results for racially and ethnically minoritized individuals. The homogenous racial identity of our sample was despite conscious attempts to recruit more Black and African American veterans living in rural Arkansas, including reaching out to Arkansas chapter members of the National Association for Black Veterans and working with established, informal liaisons in Black, rural communities in Arkansas. It is possible that adaptation results or implementation barriers and facilitators might be different among Black and African American, Hispanic and Latinx, and veterans of other racial/ethnic backgrounds. Since workgroup participants were being observed (by the research team and other participants) while conducting interviews, it is possible they may not have given their true thoughts and feedback. This was why it was important to orient the group before interviews, review the community agreements, and conduct anonymous surveys and voting during data collection.

## 5. Conclusions

Safety Planning Intervention via peer-to-peer delivery, especially in rural community settings where suicide disparities exist, was seen as very acceptable and advantageous given existing contextual barriers to spreading this effective intervention to all who need it. We identified adaptations needed for peer-to-peer delivery for this intervention and produced a document for future dissemination and training efforts for other rural states that may wish to adopt peer-to-peer Safety Planning Intervention for veterans in their communities. We also identified copious barriers and facilitators to real-world implementation of this intervention in rural communities, which can be used to inform implementation strategies and planning efforts in a thoughtful way, centering the community voice from the beginning. This study is an example of community-engaged implementation science in pre-implementation stages to promote future equitable spread and scale of an effective intervention.

## Data Availability

The raw data supporting the conclusions of this article will be made available by the authors, without undue reservation.
