# Supplementary material for: Pre-implementation adaptation of suicide safety planning intervention using peer support in rural areas
Source: Front Health Serv. 2023 Dec 22;3:1225171. doi: 10.3389/frhs.2023.1225171 (PMC10766826; doi:10.3389/frhs.2023.1225171)
Supplement: Supplementary file 2 [file Table2.docx]

Blank Template

Workgroup #:

Date of workgroup meeting:

Coder:

| **Categories** | **Responses** |
| --- | --- |
| **Possible core / adaptable intervention components:** Aspects of Safety Planning Intervention as a process or the form or information provided that could be changed (adapted) or must remain the same as it is used in healthcare settings (core)  -Were there any decisions made about intervention components?  -Is there a sense of whether those adaptations are red/yellow/green light?  (Note: log adaptations in FRAME) |  |
| **Implementation barriers or facilitators:** Factors that will make it harder (barriers) or easier (facilitators) to roll this out in community organizations after it is adapted. In other words, what people foresee as challenges to overcome to spread this intervention to rural Veterans, and what people foresee as advantages to be harnessed. |  |
| **Measures for future pilot:** metrics of success that this intervention is acceptable, feasible, and can be done with quality |  |
| **Were there any quantitative results from real-time voting?** (if yes, what were results?) |  |
| **Other notes:** Comments about other aspects of this work, such as suicide risk factors in rural areas or training needs for peer-to-peer delivery |  |
