# Supplementary material for: Pre-implementation adaptation of suicide safety planning intervention using peer support in rural areas
Source: Front Health Serv. 2023 Dec 22;3:1225171. doi: 10.3389/frhs.2023.1225171 (PMC10766826; doi:10.3389/frhs.2023.1225171)
Supplement: Supplementary file 3 [file Table3.docx]

Supplemental File. Survey

1. Do you feel comfortable sharing openly your thoughts in the group meetings we are hosting with other Veterans and members of research study?

Yes

No

<skip logic> If no, why not?

If no, are you comfortable sharing on this survey anonymously?

If no, what is another method where you would be comfortable sharing your thoughts with us for this study?

1. What are factors making you less comfortable sharing in the group?
2. Is there anything we can do to help make it more comfortable for you to share your ideas and thoughts in the group?

[open ended text box]

1. What do you think the goal of this research study is?

[open ended text box]

1. What thoughts do you have about the topic of the study: changing safety planning to be delivered outside hospitals and instead, by peers in the community, for Veterans with suicidal thoughts but not at high risk (crisis)?
   1. Response options included each step of safety planning and a rating whether they wanted to change it, keep it the same, or were unsure.
   2. If they selected change it or keep it the same, they were given an open ended text box to elaborate.
